# Where to Find Leucine in Food and How to Feed Elderly With Sarcopenia in Order to Counteract Loss of Muscle Mass: Practical Advice

**DOI:** 10.3389/fnut.2020.622391

**Published:** 2021-01-26

**Authors:** Mariangela Rondanelli, Mara Nichetti, Gabriella Peroni, Milena Anna Faliva, Maurizio Naso, Clara Gasparri, Simone Perna, Letizia Oberto, Enrica Di Paolo, Antonella Riva, Giovanna Petrangolini, Giulia Guerreschi, Alice Tartara

**Affiliations:** ^1^Istituto di Ricovero e Cura a Carattere Scientifico (IRCCS) Mondino Foundation, Pavia, Italy; ^2^Department of Public Health, Experimental and Forensic Medicine, University of Pavia, Pavia, Italy; ^3^Endocrinology and Nutrition Unit, Azienda di Servizi alla Persona “Istituto Santa Margherita”, University of Pavia, Pavia, Italy; ^4^Department of Biology, College of Science, University of Bahrain, Zallaq, Bahrain; ^5^General Geriatric Unit, Azienda di Servizi alla Persona “Istituto Santa Margherita”, Pavia, Italy; ^6^Research and Development Department, Indena SpA, Milan, Italy

**Keywords:** leucine, proteins, sarcopenia, elderly, diet

## Abstract

The term sarcopenia refers to the loss of skeletal muscle mass and strength that generally occurs during aging. The interventions that have proved most effective in reducing the severity and preventing the worsening of sarcopenia include physical exercise, especially resistance, and the administration of dietary supplements in association with a targeted diet; nutritional intervention is the main therapeutic approach for elderly people, since they are very often sedentary (also due to possible disabilities). Among the various nutrients, high biological value proteins and leucine are of particular interest for their demonstrated effects on the health of skeletal muscle. The intake of food containing proteins and leucine during meals stimulates muscle protein synthesis. Lower blood levels of leucine were associated with lower values of the skeletal muscle index, grip strength and performance. The international guidelines recommended that a leucine intake of 3 g at three main meals together with 25–30 g of protein is the goal to be achieved to counteract loss of lean mass in elderly. Food composition databases rarely show the amounts of leucine contained in foods and therefore it becomes difficult to build a diet that follows these guidelines. A table was therefore created for the first time in the literature to collect all the foods richest in leucine, thanks to the union of the most important Italian food databases. Moreover, in order to implement a diet that follows the right recommendations, another tables shows nutritional composition of breakfast, lunch and dinner (that each provide 3 grams of leucine and 25 grams of protein) for seven days.

## Introduction

The term sarcopenia refers to the loss of skeletal muscle mass and strength that generally occurs during aging (from the age of 50); in individuals aged 60–70, a prevalence of about 5–13% has been estimated, which increases to 11–50% after the age of 80, with a consequent increase in physical disability, poor quality of life and increased morbidity and mortality in this slice of the population (1, 2).

The interventions that have proved most effective in reducing the severity and preventing the worsening of sarcopenia include physical exercise, especially resistance, and the administration of dietary supplements in association with a targeted diet; nutritional intervention remains the main therapeutic approach in elderly people, since they are very often sedentary (also due to possible disabilities) (3). Among the various nutrients, high biological value proteins and leucine are of particular interest for their demonstrated effects on the health of skeletal muscle (4). The intake of food containing protein during meals stimulates muscle protein synthesis, with a peak about 2–3 h after ingestion (5). Aging is associated with muscle resistance to the anabolic effects of diet, with a reduced response to postprandial hyperaminoacidemia, in terms of muscle protein synthesis, compared to young adults (6, 7). The international guidelines recommended that a leucine intake of 3 g at three main meals together with 25–30 g of protein is the goal to be achieved to counteract loss of lean mass in elderly (8).

Food composition databases rarely show the amounts of leucine contained in foods and therefore it becomes difficult to build a diet that follows these guidelines.

Given this background, the aim of this narrative review is to evaluate the literature and the food composition databases which considered the leucine content on food, by building tables with leucine content in food, in order to prepare practical advice for sarcopenic elderly.

## Materials and Methods

The present narrative review was performed following the steps by Egger et al. (9) as follows:

1. Configuration of a working group: three operators skilled in clinical nutrition (one acting as a methodological operator and two participating as clinical operators).
2. Formulation of the revision question on the basis of considerations made in the abstract: “because food composition databases rarely show the amounts of leucine contained in foods, the aim of this narrative review is to evaluate the literature and the food composition databases which considered the leucine content on food, by building tables with leucine content in food, in order to prepare practical advice for sarcopenic elderly”.
3. Identification of relevant studies: a research strategy was planned on PubMed (Public MedIine run by the National Center of Biotechnology Information (NCBI) of the National Library of Medicine of Bathesda (USA)) as follows: (a) Definition of the keywords (leucine, food, muscle mass), allowing the definition of the interest field of the documents to be searched, grouped in quotation marks (“…”) and used separately or in combination; (b) use of: the Boolean (a data type with only two possible values: true or false) AND operator, that allows the establishments of logical relations among concepts; (c) Research modalities: advanced search; (d) Limits: time limits: papers published in the last 20 years; humans; adults; languages: English; (e) Manual search performed by the senior researchers experienced in clinical nutrition through the revision of reviews, individual articles and food composition databases.
4. The analysis was carried out in the form of a narrative review of the reports.

## Results

### The Pivotal Role of Leucine in Order to Counteract the Loss of Muscle Mass

Metabolic studies have shown that subjects ≥65 years of age require about 2% more protein per meal in order to maximize muscle protein synthesis, and leucine supplementation has been shown to be potentially useful for this purpose (3, 10). L-leucine is an essential non-polar aliphatic, branched-chain amino acid (branched-chain amino acid, BCAA, such as L-valine and L-isoleucine), with regulatory functions on the muscle mediated, at least in part, by the action of mTOR (mammalian Target Of Rapamycin, target of rapamycin in mammals, a serine-threonine protein kinase) (3, 11). MTOR, and the signal transduction pathways that originate from it, regulate protein synthesis and act as energy “sensors” of nutrients and the availability of amino acids (leucine in particular); mTOR is activated when ATP levels are high, and blocked when ATP levels are low (12). In general, BCAAs, unlike other amino acids, which are oxidized mainly in the liver, are metabolized mainly in skeletal muscle, where the most active oxidation system of branched chain amino acids is located, including the BCAT (branched-chain aminotransferase) enzyme (13). Leucine, in particular, acts directly, as a signal molecule, through the activation of the mTORC1 complex, which regulates the homeostasis of proteins by stimulating the processes of translation and muscle protein synthesis in response to amino acids (phosphorylation of the translation initiation factor 4E-BP1) and repressing protein degradation (6, 14); furthermore, its integration has appeared to improve mitochondrial function in skeletal muscles (3, 11). At the muscle level, therefore, leucine decreases proteolysis and stimulates protein synthesis, while at the level of pancreatic beta-cells it has a secretagogue effect, enhancing the glucose-induced release of insulin. Furthermore, this amino acid can stimulate the synthesis and secretion, by the L cells of the ileum and colon, of GLP-1 (Glucagon-Like Peptide 1), which increases the insulin response of muscle cells, thus improving the 'glucose intake and, consequently, anabolism and the maintenance of muscle mass itself (3, 11).

### Leucine Blood Levels and Muscle Mass and Performance

As for the plasma levels of branched chain amino acids, and therefore also of leucine, they seem to be influenced by changes in the dietary intake of nutrients. From observations on humans and animal models, for example, an increase in the concentrations of these amino acids emerged following a period of fasting, while protein deprivation was associated with a decrease. In the first case, the main responsible mechanism would be an increase in muscle protein degradation, aimed at providing substrates for hepatic gluconeogenesis and the oxidation of BCAAs in the muscles; in the second case, the absence of exogenous amino acids and the reduced muscle protein turnover would explain the decrease in plasma levels of BCAA (15). In the post-prandial period, on the other hand, a rapid increase was observed in 11 healthy male volunteers aged between 40 and 61 years, followed by the uptake of BCAA by muscle tissue, and these plasma concentrations were still elevated 12 h after a high protein meal (4,850 kJ of energy, of which 40% provided by protein, 30% by fat and another 30% by carbohydrates) compared to a low protein meal (3,770 kJ of energy: 15% deriving from proteins, 25% from fats and 60% from carbohydrates) (16). A Canadian study of 2019 on 199 subjects aged 18–55, however, found no association between fasting plasma levels of BCAAs (sample mean: 455.6 ± 92.3 μM) and their dietary intake (value average: 18.5 ± 6.8 g/day); a weak correlation was instead found between plasma concentration of branched chain amino acids and intake of proteins of animal origin and red meat (17).

In a sample of 232 elderly Japanese women (mean age 79.4 ± 7.0 years) significantly lower blood leucine concentrations were found in subjects with dinapenia (defined by the authors as decreased muscle function without loss of muscle mass) and sarcopenia compared to normal subjects. More generally, in women with dinapenia and sarcopenia the concentrations of BCAAs were lower than in normal women and women with pre-sarcopenia (reduced muscle mass without decreased muscle function) (18). Similarly, in a Norwegian cross-sectional study, conducted on 417 elderly subjects (age ≥ 70 years), after adjustment of the data for gender, it was found that the plasma concentrations of leucine and isoleucine, after a mean of 2 h and 18–24 min from the last meal, are significantly lower in participants diagnosed with sarcopenia than in healthy ones, as well as absolute dietary protein intake and energy intake assessed by two 24-h recall. Adjusting, however, the data also for body weight, no difference in protein intake was highlighted between the two groups. Furthermore, plasma leucine levels correlated positively with skeletal muscle mass and grip strength. However, it remains to be clarified whether the low dietary intake of proteins over time is the cause of the low plasma concentrations of BCAAs, and whether the levels of leucine in particular can be used as biomarker of sarcopenia; in fact, to date, no optimal or minimum plasma values necessary for the maintenance of skeletal muscle mass have been defined for branched chain amino acids (19). Furthermore, using data from the Maastricht Sarcopenia study (MaSS) cross-sectional study of 227 community-resident seniors (mean age 74 years), Ter Borg S. and colleagues observed lower blood levels of essential amino acids, branched chain amino acids and leucine were associated with lower values of the skeletal muscle index (SMI) and grip strength and a longer time required to complete the chair stand test; moreover, the condition of sarcopenia was more frequent among subjects with lower levels of leucine, BCAAs and essential amino acids (EAAs) (20). Moreover, some comparative studies have investigated the differences in the plasma amino acid profile of healthy individuals belonging to different age groups. Comparing between them 27 young subjects (10 males of 26.3 ± 0.8 years and 17 females of 25.0 ± 0.6 years), 40 elderly (20 males of 75.8 ± 0.5 years and 20 females of 76.3 ± 0.8 years) and 32 centenarians (nine males of 101.9 ± 1.0 years and 23 females of 102.0 ± 0.5 years), Chan YC and colleagues found levels of branched chain amino acids (BCAAs) gradually lower with advancing age in both sexes (21). Similar results had already been reported in 1989 by Rudman D. et al., who studied fasting plasma levels of amino acids in four groups of individuals: 22 healthy young men (25–35 years), 21 healthy elderly men and self-employed (65–85 years), 23 institutionalized men with dementia, fed orally (65–92 years) and 17 institutionalized men with dementia, but artificially fed (65–88 years). The last three groups had shown significantly lower levels of BCAAs than the first group (22). And again, in another study, evaluating the amino acid concentrations in 72 healthy subjects, aged 23–92 years, divided into six groups of 12 participants each based on sex and age (20–39, 40–59 and >60 years), it was observed that the levels of total amino acids, EAAs and non-essential and branched chain amino acids decreased with increasing age; furthermore, men showed significantly higher concentrations of EAAs and BCAAs than women. Based on these data, the authors suggest that the reduction in serum amino acid concentrations may represent a consequence of the reduced energy and protein intake (evaluated in this study through a 5-day food diary) that often occurs during aging (23). Dietary intake of amino acids and its possible correlation with the skeletal muscle mass index (SMI) were evaluated in a recent Korean study in a sample of adults aged 50–64 years. The intake of leucine, isoleucine and valine (estimated through a 24-h recall) was found to satisfy the recommended values for the Korean population and a significant positive association emerged between the intake of BCAAs with the diet (in g/day) and the SMI (24).

### Intervention Study With Leucine in Sarcopenic Elderly

Most of the clinical intervention studies have been performed on subjects residing in the community and using, as a source of leucine, whey proteins, which are particularly rich in it (approximately 13 g of leucine/100 g of protein), while only in a few studies was supplementation with leucine alone used (3).

In a randomized controlled study conducted in Spain of 42 institutionalized elderly individuals (mean age 78.9 ± 7.9 years; 14 men and 29 women), the administration of 6 g/day of L-leucine for 13 weeks improved performance. functional (assessed through the measurement of pace speed) and the strength of the respiratory muscles (increase of the maximum static expiratory pressure measured at the mouth, MEP), and prevented the decline of the lean mass index (assessed through bioimpedance analysis, BIA) compared to placebo, suggesting beneficial effects of L-leucine supplementation on sarcopenia in elderly subjects. In this population, in fact, leucine would help maintain muscle mass stable over time and improve nutritional status (3). Katsanos had also observed how, while in young adults both the intake of a mixture of 6.7 g of essential amino acids (EAAs) containing 26% of leucine (equal to about 1.7 g), both intake of an equal amount of EAAs containing 41% of this amino acid (equal to about 2.8 g) were able to increase muscle protein synthesis, in elderly individuals this effect would occur only with the mixture containing the higher % of leucine (25). A 2018 randomized controlled trial showed that both the presence of leucine (3 g twice a day), and not the total amount of protein (10 g vs. 25 g, twice a day), is provided by supplementation for 6 days, the main responsible for the increase in myofibrillar protein synthesis in a sample of healthy women aged 65–75 years (26). The same authors had already shown, in a previous study conducted on the same sample, how the integration with 15 g of a protein drink containing 4.2 g of leucine, taken twice a day for 6 days, caused a greater increase in synthesis myofibrillar protein compared to using 15 g of a similar drink, but containing only 1.3 g of leucine (7). Even in healthy elderly men (65–85 years of age) the intake of 5 g of leucine at each of the three main meals, for 3 days, improved myofibrillar protein synthesis, both in subjects with dietary protein intake equal to 0, 8 g/kg of body weight/day, as in those with a dietary intake of 1.2 g of protein/kg/day (27). From a 2015 meta-analysis, performed on 16 randomized controlled or cross-sectional studies with a duration ranging from 10 days to 2 years, it emerged that leucine supplementation in a total of 999 elderly individuals (2 to 7.8 g/day leucine, administered via amino acid mixtures, whey protein, casein, cottage cheese or protein-enriched energy drinks) was associated with a significant increase in body weight and lean mass, particularly in the subgroup of subjects diagnosed with sarcopenia; however, no effect on muscle strength was found (28). Another meta-analysis was conducted, in the same year, on 228 elderly individuals, selecting nine randomized clinical trials: of these, four investigated the acute effects of a single administration of leucine (from 2.6 to 17.6 g/day), while the remaining five were based on longer supplementation periods (10 days to 6 months; leucine between 2.8 and 16.1 g/day). Overall, the administration of leucine (also in this case in the form of various preparations, pure or in combination with other amino acids) was associated with a significant increase in the rate of muscle protein synthesis, but not in lean body mass and lower limbs (14). The heterogeneity of the studies analyzed makes it difficult to draw definitive conclusions, but it is plausible to admit a beneficial effect of supplementation with leucine in subjects at risk or suffering from sarcopenia (4).

### Recommended Amount of Leucine Intake

In elderly individuals, the recommended amount of protein intake is 1.0–1.2 g/kg of body weight/day, such as to maintain a positive protein status, thus obviating the altered anabolic response to proteins typical of aging; however, in the US NHANES study, it was found that up to 46% of the elderly population does not reach the average daily protein intake, recommended for adults, of 0.8 g/kg body weight (29, 30). In particular, then, according to the World Health Organization (WHO), for a healthy adult the daily requirement of BCAA is equal to 39 mg/kg of body weight for leucine, 20 mg/kg for isoleucine and 26 mg/kg for valine (with no specific differences for the elderly population) (31). Furthermore, the directives of the Italian Ministry of Health had established that the intake of branched chain amino acids should not exceed 5 g/day (as the sum of the three) and suggest a ratio of 2:1:1 between leucine, isoleucine and valine, respectively (32).

Based on the analysis of the data reported in the literature, the PROT-AGE Study Group has proposed, for elderly individuals, an anabolic threshold of protein intake equal to 25–30 g of protein per meal, containing about 2.5–2.8 g of leucine, therefore higher than that for young adults (29).

### Leucine in Foods and Practical Advice

Leucine is a ubiquitous amino acid, present in all proteins, although more abundant in those of animal origin; it is therefore practically impossible to separate its intake from the total protein intake through the diet (4).

The recommended intake of 3 g of leucine at the three main meals together with 25–30 g of protein is therefore the goal to be achieved to prevent or recover the loss of lean mass in the elderly (29). Food composition databases rarely show the amounts of leucine contained in foods and therefore it becomes difficult to build a diet that follows these guidelines.

## Conclusions

A table (Table 1) was therefore created for the first time in the literature in order to collect all the foods richest in leucine, thanks to the union of the two most important Italian food databases: Banca dati di composizione degli alimenti- istituto europeo di oncologia (BDA-IEO) (33) and consiglio per la ricerca in agricoltura e l'analisi dell'economia agraria (CREA) (34). The completeness of the table allows a wide choice of foods, thanks to which the amount of leucine necessary for the purpose is more easily reached.

**Table 1:** Content in leucine, protein and energy per 100 g of food (in descending order of leucine).

**Food**	**Leucine (g/100 g)**	**Total protein (g/100 g)**	**Energy (kcal/100 g)**
**Meat**			
Adult bovine's rump	1,894	22	111
Baked ham	1,695	19.8	215
Bovine, calf, 4 months, low-fat meat	1,029	20.7	92
Bovine, calf, 4 months, semi-fat meat	1,742	20.3	144
Bresaola	2,651	32	152
Chicken's breast, without skin	1,955	23.3	100
Chicken's wings, without skin	1,717	20.3	193
Deer, without visible fat	1,953	21	91
Guinea fowl's breast, without skin	2,180	25,8	121
Guinea fowl's thigh, without skin	1,829	24	127
Horse, fat and muscle tissue	1,519	19,8	145
Lamb	1,532	20	159
Melted chicken, without skin	1,526	18.5	107
Pork low-fat steak, without visible fat	1,741	21.3	157
Pork's sausage	1,241	15,4	304
Pork's shoulder	1,550	19	156
Rabbit, low-fat meat	1,987	23.7	102
Raw ham	2,211	26.6	284
Speck	2,326	28,3	303
Turkey's breast, without skin	2,002	24	107
Turkey's thigh, without skin	1,438	18	113
**Cheese, milk and yogurt**			
Asiago	2,845	31.4	359
Cow ricotta	0,997	8.8	146
Cow's milk partially skimmed	0,377	3.5	46
Crescenza	1,250	16.1	281
Emmenthal	2,687	28.5	403
Feta	1,531	15,6	250
Gorgonzola	1,530	19,1	324
Grana cheese	2,820	33.9	406
Greek yogurt	0,505	6.4	115
Greek yogurt, low-fat	0,707	9	51
Gruyer	3,184	30.6	389
Italico	2,071	21.2	316
Milk flakes	0,978	9.7	115
Mozzarella cheese	1,400	18.7	253
Parmesan	2,880	33.5	387
Robiol	1,467	20	338
Spreadable cheese	0,933	8.6	313
Yogurt partially skimmed	0,268	3.4	43
**Fish**			
Anchovy	1,331	16,8	96
Clam	0,718	10.2	72
Cod	1,484	17	71
Cod steaks	0,862	11	191
Cuttlefish	0,985	14	72
Dog fish	1,300	16	80
Drained tuna in oil	2,029	25.2	192
Farmed sea bream, filets	1,557	19.7	159
Frozen shrimps	1,179	13.6	63
Grouper	1,455	17.9	80
Herring	1,341	16.5	216
Mackerel	1,636	17	170
Mullet roe	2,822	35,5	373
Mussel	0,824	11,7	84
Octopus	0,746	10,6	57
Persic fish	1,252	15.4	75
Salmon	1,496	18.4	185
Sardines	1,643	20,8	129
Smoked salmon	2,065	25.4	147
Soaked cod	1,886	21.6	95
Sole	1,336	16.9	86
Squid	0,886	12,6	68
Surimi	1,204	15.2	95
Sword fish	1,373	16.9	109
Trout	1,028	14.7	86
Tuna fish	1,871	21.5	159
**Legumes**			
Beans	0,488	6,4	104
Borlotti beans dried, boiled	0,563	6,9	106
Borlotti beans, boiled	0,493	5,7	78
Cannellin beans dried, cooked, boiled	0,682	8	107
Cannellin beans, canned, drained	0,513	6	86
Chick peas dried, boiled	0,549	7	132
Lentils, drained canned	0,417	5	91
Lentils, dried boiled	0,527	6,9	109
Peas, dried	1,406	21,7	306
Raw dried broad beans	2,119	27,2	343
**Cereals**			
Bread	0,691	9	275
Buckwheat	0,837	12,4	329
Corn	1,168	9,2	357
Corn flour	1,028	8.7	341
Millet	1,389	11,8	343
Oat flour	0,920	12,6	378
Polished rice	0,590	6,7	334
Rusks	0,831	11,3	387
Semolina pasta	1,033	13,5	341
Spelled perlat, raw	1,075	14,6	353
**Dried fruit**			
Cashew nuts	1,280	15	604
Dried fruit	0,848	12.9	660
Hazelnuts, dried	0,930	13.8	625
Nuts, dried	1,011	14.3	702
Pine nuts	2,054	31,9	604
Pistachios	1,442	20,6	570
Sweet almonds, dried	1,450	22	542
**Fruit**			
Ananas	0,022	0,5	40
Apple with peel	0,012	0.2	44
Apricot	0,022	0,4	28
Avocado	0,315	4.4	238
Banana	0,056	1.2	76
Black cherry	0,023	0,8	41
Bluebarry	0,054	0.9	49
Cestnut	0,207	3,5	189
Cherry	0,023	0,8	38
Fig	0,04	0,9	47
Grapes	0,014	0,5	61
Kiwi	0,068	1,2	44
Melon	0,028	0,8	33
Orange	0,022	0.7	37
Peach	0,029	0,8	27
Pear	0,016	0,3	35
Raspberry	0,051	1	34
Strawberry	0,046	0,9	27
**Vegetables**			
Artichokes	0,196	2,7	22
Chard	0,093	1,3	17
Coultivated mushrooms, pleurotes	0,172	2,2	37
Eggplant	0,070	1,1	15
Forest asparagus	0,210	4,6	35
Fresh lettuce	0,115	1,8	19
Fresh ripe tomatoes	0,030	1	19
Green beans	0,147	2,1	18
Green cabage	0,113	2,1	19
Pepperoni	0,039	0,9	25
Porcini mushrooms	0,207	3,9	27
Spinaches	0,323	3,4	31
Zucchini/courgettes	0,130	1,3	11
**Other**			
Arachid butter	1,465	22.6	623
Butter	0,086	0,8	758
Chicken egg	1,041	12.4	128
Chicken egg white	0,862	10,7	43
Potatoes	0,122	2,1	85
Sweetened cocoa soluble powder	0,273	4.5	349
Unsweetened cocoa powder	1,238	20.4	355

In order to make it easy in order to implement a diet that follows the recommendations previously provided, Table 2 shows nutritional composition of breakfast, lunch and dinner (that each provide 3 grams of leucine and 25 grams of protein) for 7 days.

**Table 2:** Nutritional composition of breakfast, lunch and dinner for each day for a week.

	**Breakfast**	**Lunch**	**Dinner**	**All day**
**Monday**				
Meal/Recipe	Low-fat milk (300 g) and rusks (30 g) with peanut butter (30 g) and fresh fruit (kiwi 150 g)	Pasta (100 g) with swordfish (80 g) and mintSalad (50 g) with cherry tomatoes (200 g) Greek yogurt (125 g) with dried sweet almonds (10 g), bitter cocoa (5 g) and banana (125 g)	Baked zucchini omelet (two eggs, zucchini 100 g, courgette flowers 2/3, Grana Padano 10 g, olive oil 5 mL, salt, pepper, thyme to taste) Toasted bread (100 g) with spinaches (200 g) Peach (150 g) with bitter cocoa (5 g)	
Nutritional composition	E: 507 kcal (2,122,3 kJ), P: 22 g, L: 1,9 g	E: 841 kcal (3,520,43 kJ), P: 42.7, L: 3,15 g	E: 681 kcal (2,850,67 kJ), P: 37,6, L: 3,12 g	E: 2,059 kcal (8,618,97 kJ), P: 101,32 g, Li: 31,42%, CHO: 49,5%, S: 13,5%, F: 27,08 g, L: 8,191 g
**Tuesday**				
Meal/Recipe	Bread (50 g) with bresaola (50 g) accompanied with whole Greek yogurt (150 g) and fruit (kiwi 100 g)	Fregola with lentils (Fregola 80 g, dry boiled lentils 100 g, ripe tomatoes about 100 g, Grana Padano 20 g, extra virgin olive oil 5 mL, vegetable broth to taste, salt to taste) Zucchini (200 g) Partially skimmed white yogurt (125 g) with honey (10 g) and dried nuts (20 g)	Beef with grilled vegetables (Beef filet 120 g, eggplant 100 g, zucchini 100 g, potatoes 350 g, olive oil 5 mL, vinegar to taste, salt, parsley, basil to taste) Rice crackers (2: 16 g) Strawberries (50 g), peach (50 g), banana (50 g)	
Nutritional composition	E: 369,7 kcal (1,547,56 kJ), P: 31,3 g, L: 2,5 g	E: 773,6 kcal (3,238,29 kJ), P: 35,2 g, L: 2,744 g	E: 752,8 kcal (3,151,22 kJ), P: 34,4 g, L: 2,698 g	E: 1,976 kcal (8,271,54 kJ), P: 102,77 g, Li: 32,01%, CHO: 47,07%, S: 10,24%, F: 28,72 g, L: 7,941 g
**Wednesday**				
Meal/Recipe	Pancake (partially skimmed milk 200 g, oat flour 50 g, two egg whites: 60 g) with hazelnut cream (toasted hazelnuts 45 g, 1 tablespoon of Stevia or other sweetener, bitter cocoa powder 4 g, 1 mL seed oil) and banana (150 g) filling	Pasta with agretti and ricotta (100 g semolina pasta, 100 g cow's milk ricotta, 200 g agretti, 5 g grated parmesan, 5 mL extra virgin olive oil, fresh chili pepper to taste, a clove of garlic) Partially skimmed white yogurt (125 g) with sour cherries (150 g)	Grilled tuna with orange scent (Tuna filet 100 g, extra virgin olive oil 5 mL, rosemary to taste, a clove of garlic, juice of one orange) Potatoes (350 g), radishes (200 g) and rice crackers (2: 16 g)	
Nutritional composition	E: 758,1 kcal (3,173,41 kJ), P: 28,5 g, L: 2,28 g	E: 716,55 kcal (2,999,48 kJ), P: 30,35 g, L: 2,54 g	E: 638,42 kcal (2,672,43 kJ), P: 31,87 g, L: 2,41 g	E: 2,053 kcal (8,593,86 kJ), P: 90,33 g, Li: 31,39%, CHO: 50,86%, S: 15,13%, F: 29,85 g, L: 7,229 g
**Thursday**				
Meal/Recipe	French toast with avocado, pine nuts, eggs and grain flakes (one boiled egg 60 g, ½ avocado 100 g, bread 50 g, parmesan 30 g, pine nuts 15 g)	Risotto with shrimps, asparagus, lemon and thyme (Polished rice 100 g, shrimps 100 g, asparagus 100 g, shallot to taste, juice of ½ lemon, two sprigs of thyme, chives, salt, pepper to taste, vegetable broth to taste) Beets (200 g) Green apple sorbet with pistachio grain (one green apple about 200 g, sugar about 10 g, pistachios 30 g, water half a glass, lemon juice to taste)	Chicken strips with peppers (Chicken breast 130 g, yellow and red sweet peppers 300 g, onion 20 g, white wine half a glass to evaporate 70 mL, olive oil 10 mL, salt to taste) Bread (preferably wholemeal) (120 g) Strawberries (150 g)	
Nutritional composition	E: 593,9 kcal (2,486,07 kJ), P: 31,3 g, L: 2,45 g	E: 854,5 kcal (3,576,94 kJ), P: 39,34 g, L: 2,621 g	E: 675 kcal (2,825,55 kJ), P: 44,8 g, L: 3,557 g	E: 2,052 kcal (8,589,67 kJ), P: 104,15 g, Li: 29,65%, CHO: 49,94%, S: 12,99%, F: 27,21 g, L: 8,628 g
**Friday**				
Meal/Recipe	Low-fat milk (150 g) and toast (bread, 50 g) with smoked salmon (60 g), cream of cheese (25 g) and avocado (50 g)	Spelled with peas, asparagus and saffron (100 g spelled, 30 g dried peas, 100 g asparagus, 30 g feta, saffron in pistils, 5 mL extra virgin olive oil, salt to taste) Eggplants (200 mL) Partially skimmed white yogurt (125 g) with chopped hazelnuts (10 g) and cornflakes (30 g)	Turkey scaloppine with kiwi (Turkey breast 110 g, one kiwi 80 g, olive oil 5 mL, valerian 80 g, flour to taste, lemon juice to taste, salt to taste) Bread (preferably wholemeal) (120 g) Boiled chestnuts (45 g)	
Nutritional composition	E: 492,2 kcal (2,060,35 kJ), P: 29,34 g, L: 2,46 g	E: 783,45 kcal (3,279,52 kJ), P: 40,79 g, L: 3,133 g	E: 629,8 kcal (2,636,34 kJ), P: 41,4 g, L: 3,179 g	E: 1,933 kcal (8,091,54 kJ), P: 106,42 g, Li: 26,44%, CHO: 50,64%, S: 8,54%, F: 32,63 g, L: 8,772 g
**Saturday**				
Meal/Recipe	Mini-pancakes with oat flour (40 g oat flour, 150 g egg white, baking powder), honey (5 g) and raspberries (150 g) and low-fat milk (200 mL) with bitter cocoa (5 g)	Risotto with sausage and spinaches (100 g polished rice, 70 g pork sausage, 40 g fresh raw spinach, 20 g Parmesan cheese, 5 mL extra virgin olive oil, vegetable broth, white wine to taste, salt, pepper, nutmeg to taste) Artichokes (200 g) Apple (150 g)	Chickpeas balls with paprika (Dried boiled chickpeas 150 g, one small potato about 80 g, Grana Padano 20 g, olive oil 5 mL, breadcrumbs to taste, eggs 30 g (half egg), sweet paprika, salt, pepper to taste) Potatoes (270 g), toasted bread (20 g), lettuce (80 g) Peach (150 g)	
Nutritional composition	E: 413,4 kcal (1,730,49 kJ), P: 31,66 g, L: 2,6 g	E: 797,2 kcal (3,337,08 kJ), P: 31,05 g, L: 2,562 g	E: 796,4 kcal (3,333,73 kJ), P: 35,28 g, L: 2,611 g	E: 2,041 kcal (8,543,63 kJ), P: 93,38 g, Li: 31,15%, CHO: 50,49%, S: 11,12%, F: 45,22 g, L: 7,773 g
**Sunday**				
Meal/Recipe	Cup with milk flakes (150 g) and bitter cocoa (10 g), oat (30 g), fresh fruit (banana 100 g, blueberries 100 g, and splashed nuts (30 g)	Polenta with gorgonzola and sautéed mushrooms (100 g corn flour, 50 g gorgonzola, 200 g mushrooms, 10 g parmesan, 5 mL extra virgin olive oil, one clove garlic, salt, pepper, parsley to taste) Green beans (200 g)Kiwi (80 g), banana (80 g), blueberries (20 g)	Sorrentina sole (Sole 180 g, tomato puree approximately 100 g, one clove garlic, pitted black olives 15 g, olive oil 5 mL, flour to taste, salt, pepper, oregano, basil to taste) Rice (30 g), potatoes (300 g), fennels (200 g) Orange (150 g)	
Nutritional composition	E: 491 kcal (2,055,33 kJ), P: 28,5 g, L: 2,28 g	E: 804,4 kcal (3,367,22 kJ), P: 32,4 g, L: 2,823 g	E: 683,8 kcal (2,862,39 kJ), P: 43,3 g, L: 3,011 g	E: 2,119 kcal (8,870,13 kJ), P: 103,66 g, Li: 33,17%, CHO: 47,36%, S: 12,17%, F: 44,44 g, L: 8,113 g

*CHO, carcohydrates; E, energy; F, fiber; L, leucine; Li, lipids; P, proteins; S, sugars*.

## Author Contributions

MR designed and wrote the paper. MNi, GaP, MF, LO, GG, and AT wrote and edited the paper. MNa, CG, ED, AR, GiP, and SP visualized the paper. All authors read and approved the final manuscript.

## Conflict of Interest

AR and GiP were employed by Indena SpA. The remaining authors declare that the research was conducted in the absence of any commercial or financial relationships that could be construed as a potential conflict of interest.
